# Supplementary material for: Spatiotemporal relative risk distribution of porcine reproductive and respiratory syndrome virus in the United States
Source: Front Vet Sci. 2023 Jun 29;10:1158306. doi: 10.3389/fvets.2023.1158306 (PMC10340085; doi:10.3389/fvets.2023.1158306)
Supplement: Supplementary file 1 [file Data_Sheet_1.pdf]

Supplementary Material

Spatiotemporal relative risk distribution of porcine reproductive and respiratory syndrome virus in the United States

Felipe Sanchez[1,2], Jason A. Galvis[1], Nicolas Cardenas[1], Cesar Corzo[3], Christopher Jones[2], Gustavo Machado[1,2*]

*** Correspondence:** Gustavo Machado, gmachad@ncsu.edu

S1. Spatial relative risk - Symmetric and Asymmetric adaptive smoothing

Asymmetric adaptive smoothing is achieved by calculating separate pilot bandwidths for cases and controls using point patterns constructed from case and control farm geolocations, and calculating the global bandwidth from the entire farm population. The asymmetric approach uses different pilot bandwidths generated from disproportionate numbers of cases to controls, which has been shown to produce artifacts in the calculation of RR (Davies et al., 2016). We contrast this approach with a symmetric adaptive smoothing approach, in which the pilot and global bandwidths are determined from a point pattern constructed from combined case-control data to account for the high spatial heterogeneity of farm density in our study area (Davies and Lawson, 2019; Davies et al., 2018, 2016; Davies and Hazelton, 2010; Lawson and Zhou, 2005; Prince et al., 2001; Keeling et al., 2001).

Comparing symmetric adaptive smoothing and asymmetric adaptive smoothing, our results show that the maximum distance the spatial PRRSV RR extended to was, on average, 11.9 km for both the annual and PRRSV seasons using the symmetric adaptive smoothing, and 14.8 km for asymmetric adaptive smoothing. Both symmetric and asymmetric approaches identified similar areas of significant risk of PRRSV (Supplementary Material Figure S1 - S6), with slight variations noted for the year 2019 (Supplementary Material Figure S2), and PRRSV season 2018 - 2019 (Supplementary Material Figure S5). Areas of significant high risk generated by the symmetric approach for both the year 2019 and PRRSV season 2018 - 2019 showed several additional areas of significant high risk as compared to the asymmetric approach. However, these areas were very small (0.69 km^2); therefore, should be interpreted with caution (Davies et al., 2018, 2016) given that these areas correspond to areas of low farm density (e.g., 1 - 5 farms per 5 km^2). While the asymmetric approach has been shown to produce artifacts in the calculation of RR as compared to the symmetric approach (Davies et al., 2016), we consider the asymmetric approach to be more reasonable since it is less impacted by the uneven spatial distribution of cases to controls (Davies et al., 2018, 2016).

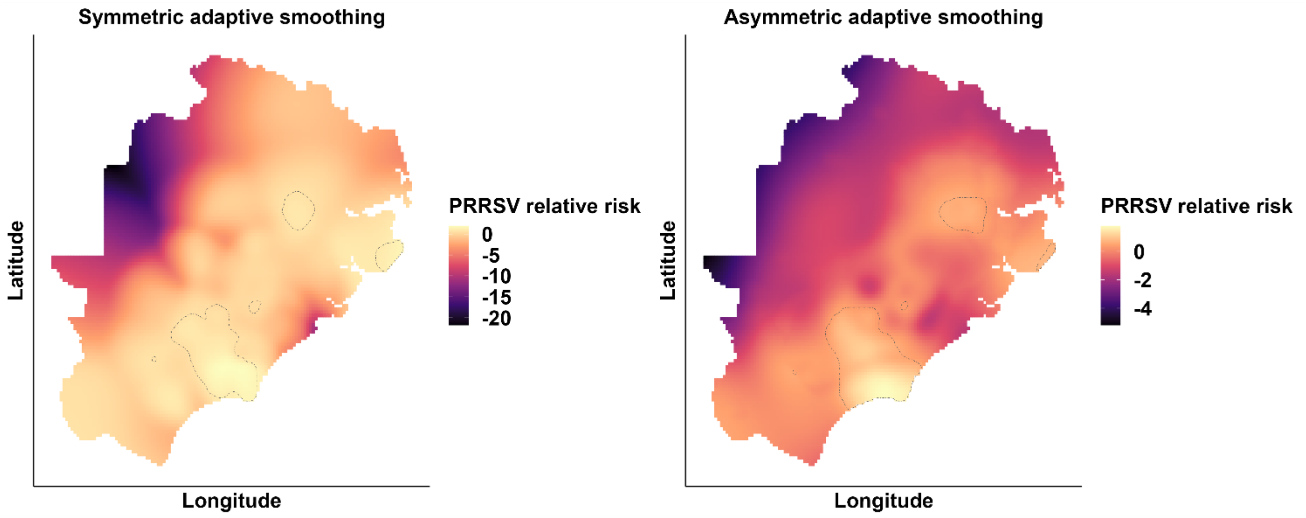

Supplementary Material Figure S1. Spatial relative risk estimates for 2018 using symmetric adaptive smoothing and asymmetric adaptive smoothing with 0.05 high risk tolerance contours denoted by a grey dash dot line.

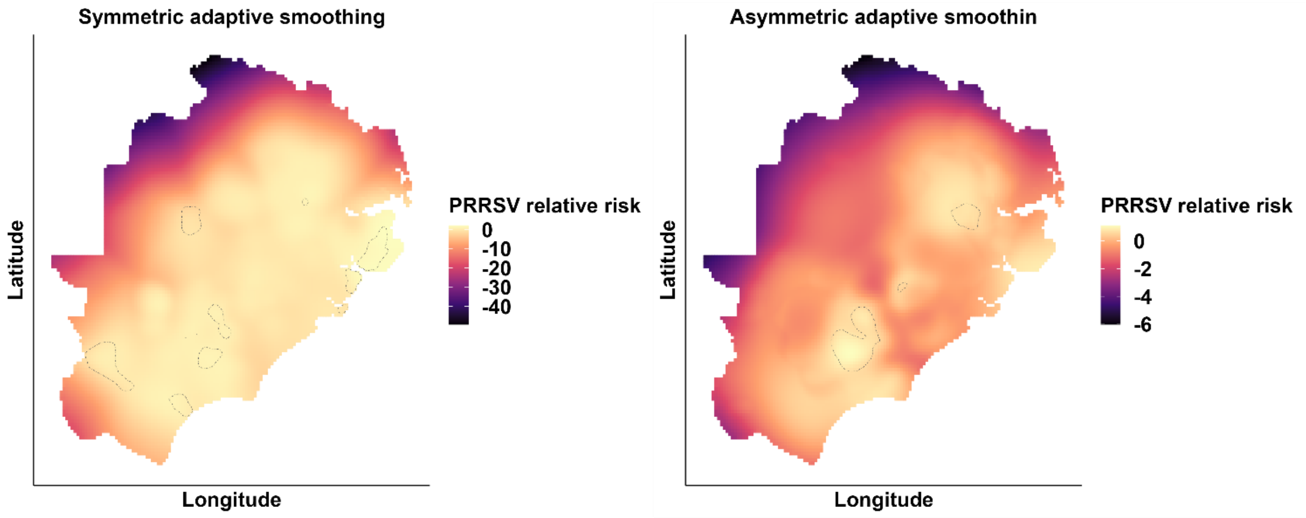

Supplementary Material Figure S2. Spatial relative risk estimates for 2019 using symmetric adaptive smoothing and asymmetric adaptive smoothing with 0.05 high risk tolerance contours denoted by a grey dash dot line.

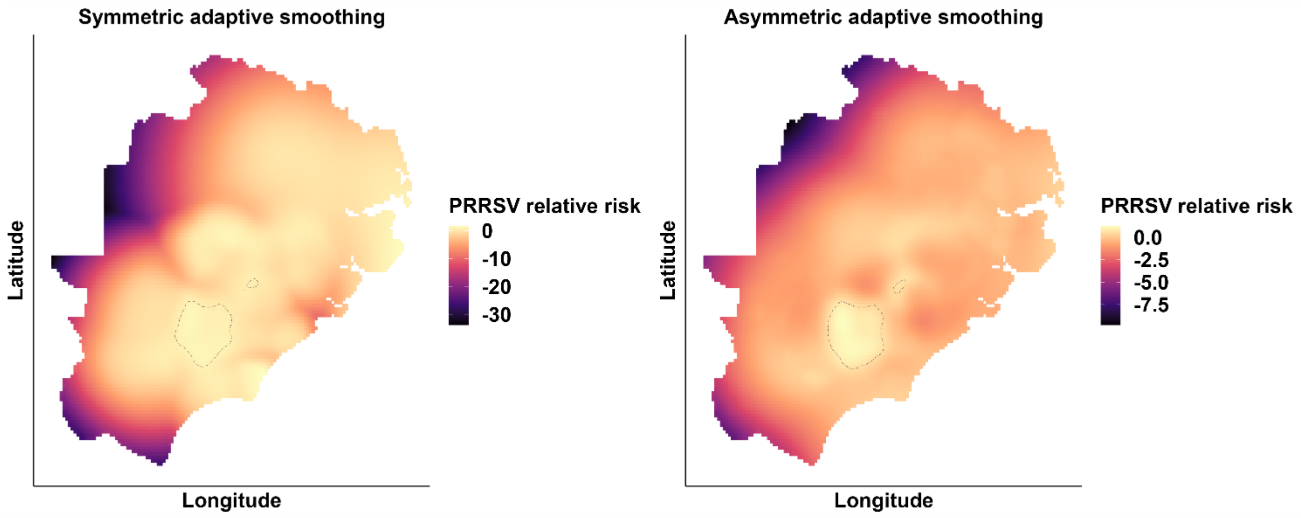

Supplementary Material Figure S3. Spatial relative risk estimates for 2020 using symmetric adaptive smoothing and asymmetric adaptive smoothing with 0.05 high risk tolerance contours denoted by a grey dash dot line.

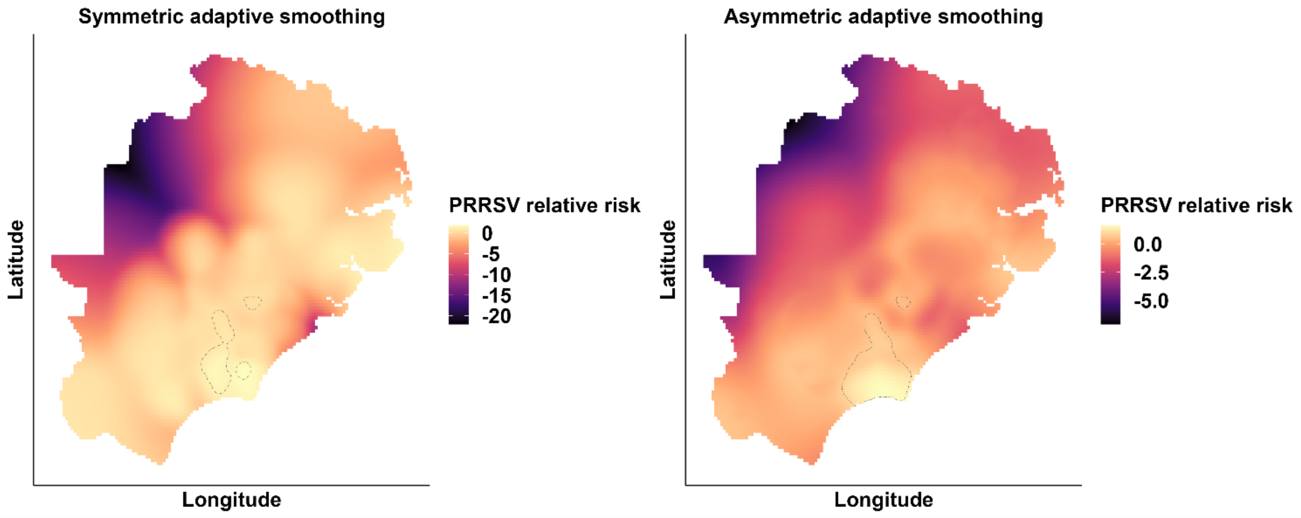

Supplementary Material Figure S4. Spatial relative risk estimates for the 2017 - 2018 PRRSV season using symmetric adaptive smoothing and asymmetric adaptive smoothing with 0.05 high risk tolerance contours denoted by a grey dash dot line.

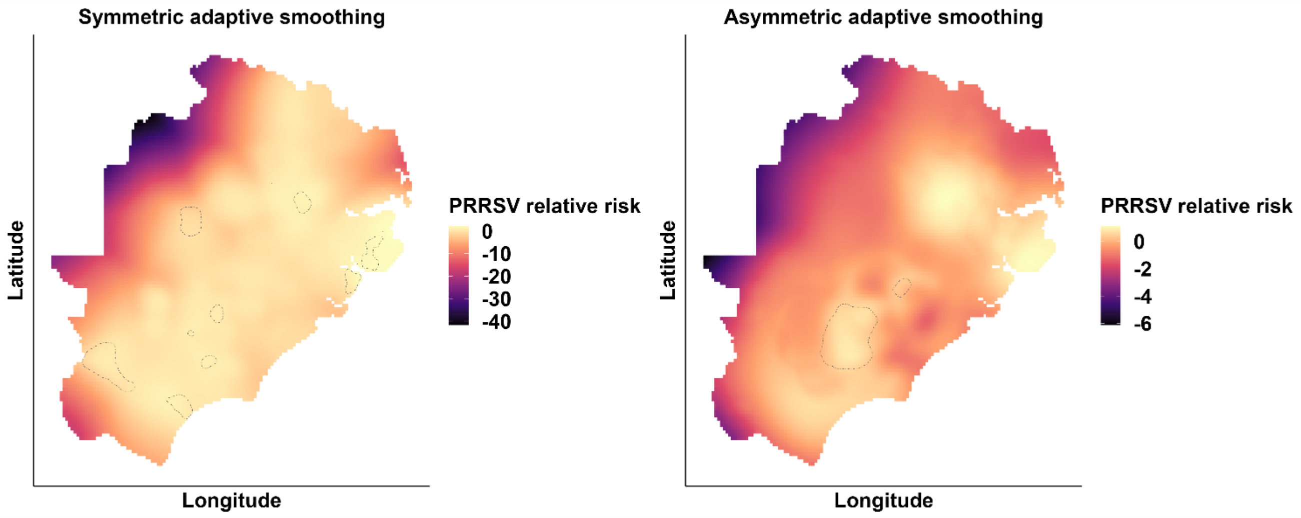

Supplementary Material Figure S5. Spatial relative risk estimates for the 2018 - 2019 PRRSV season using symmetric adaptive smoothing and asymmetric adaptive smoothing with 0.05 high risk tolerance contours denoted by a grey dash dot line.

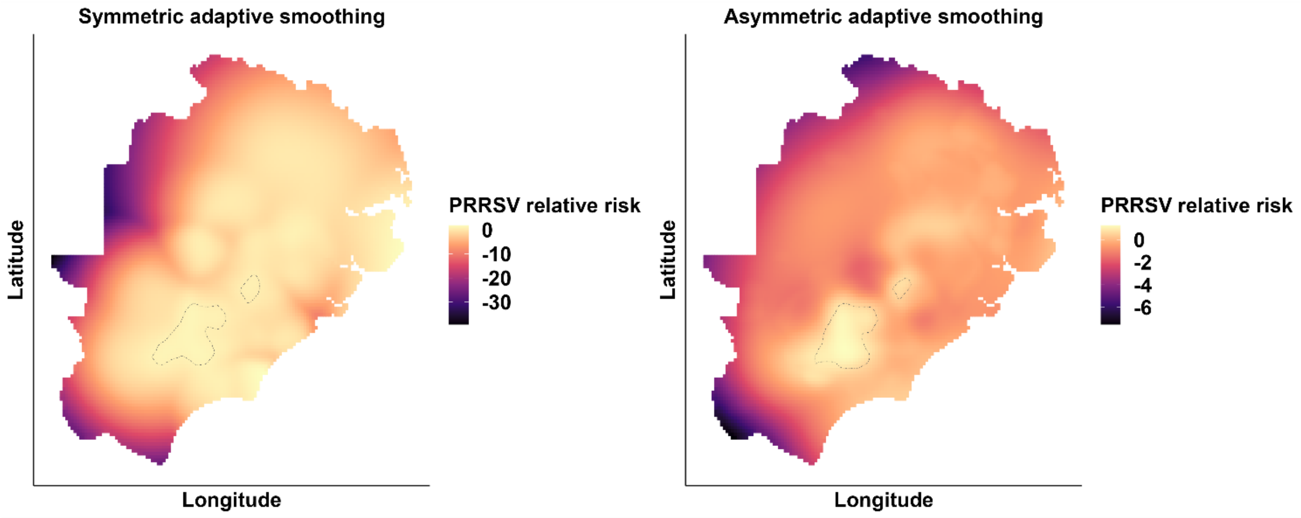

Supplementary Material Figure S6. Spatial relative risk estimates for the 2019 - 2020 PRRSV season using symmetric adaptive smoothing and asymmetric adaptive smoothing with 0.05 high risk tolerance contours denoted by a grey dash dot line.

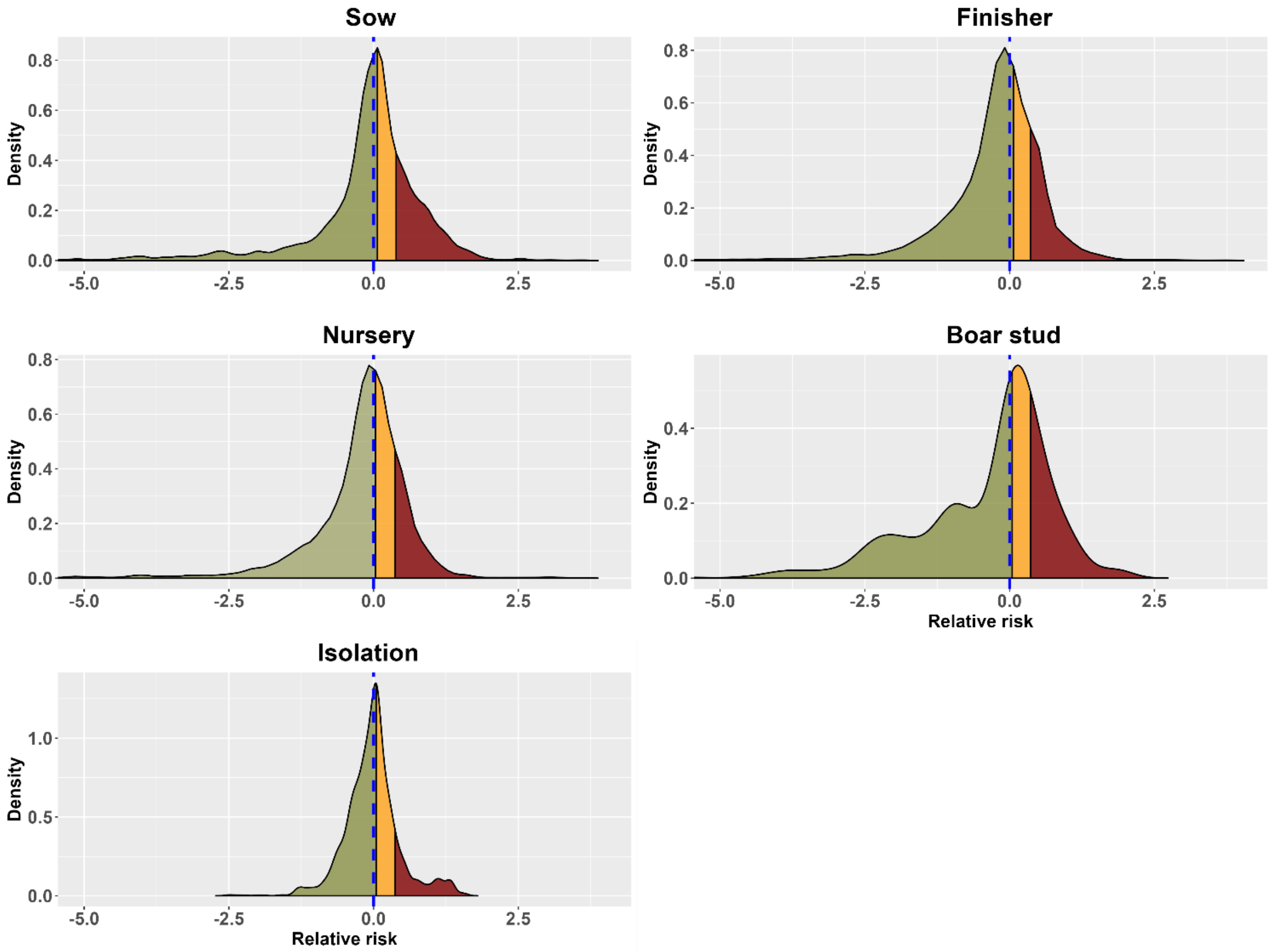

Supplementary Material Figure S7. Density plot of the spatiotemporal distribution of relative risk values for the year 2018.

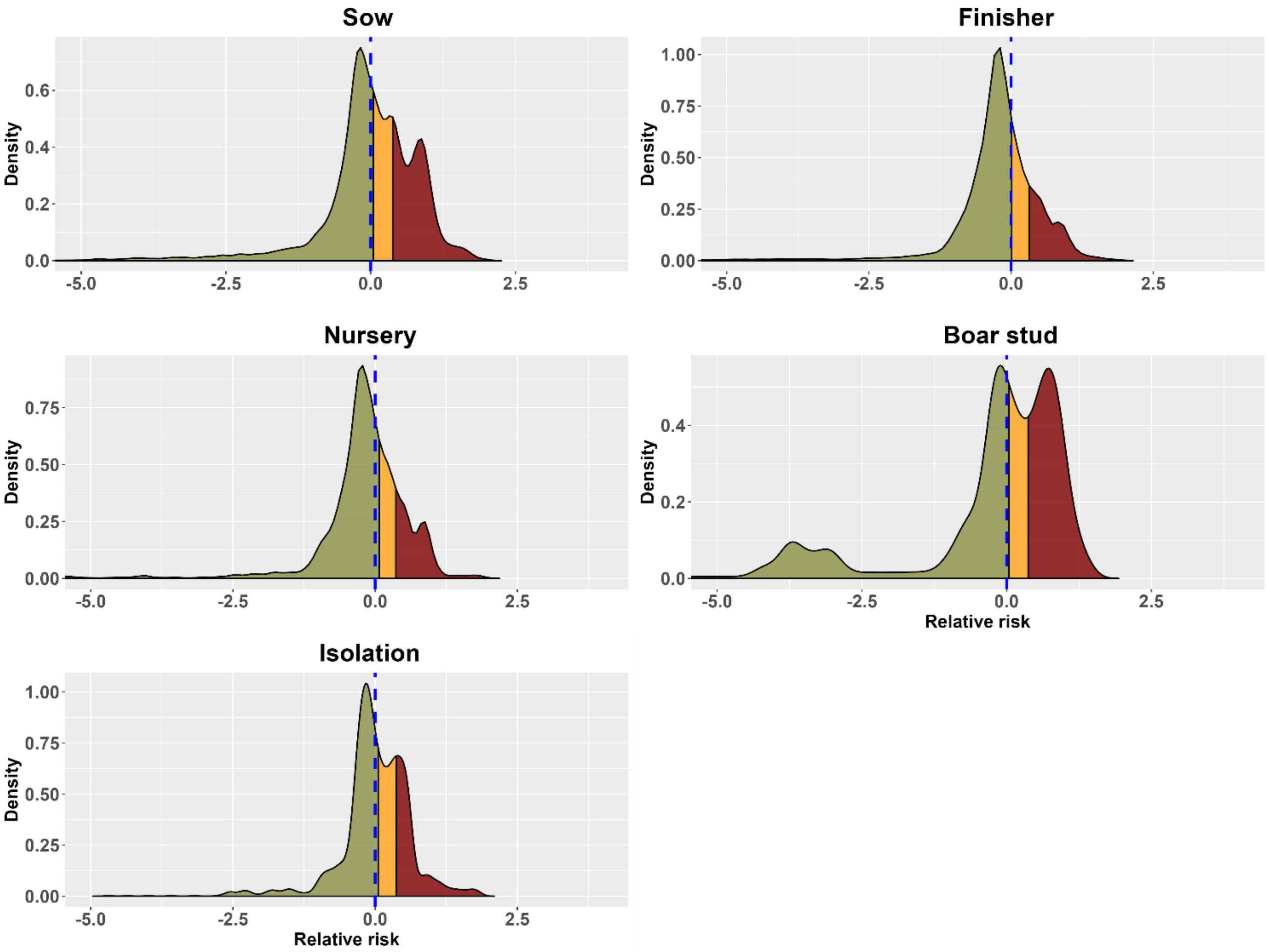

Supplementary Material Figure S8. Density plot of the spatiotemporal distribution of relative risk values for the year 2019.

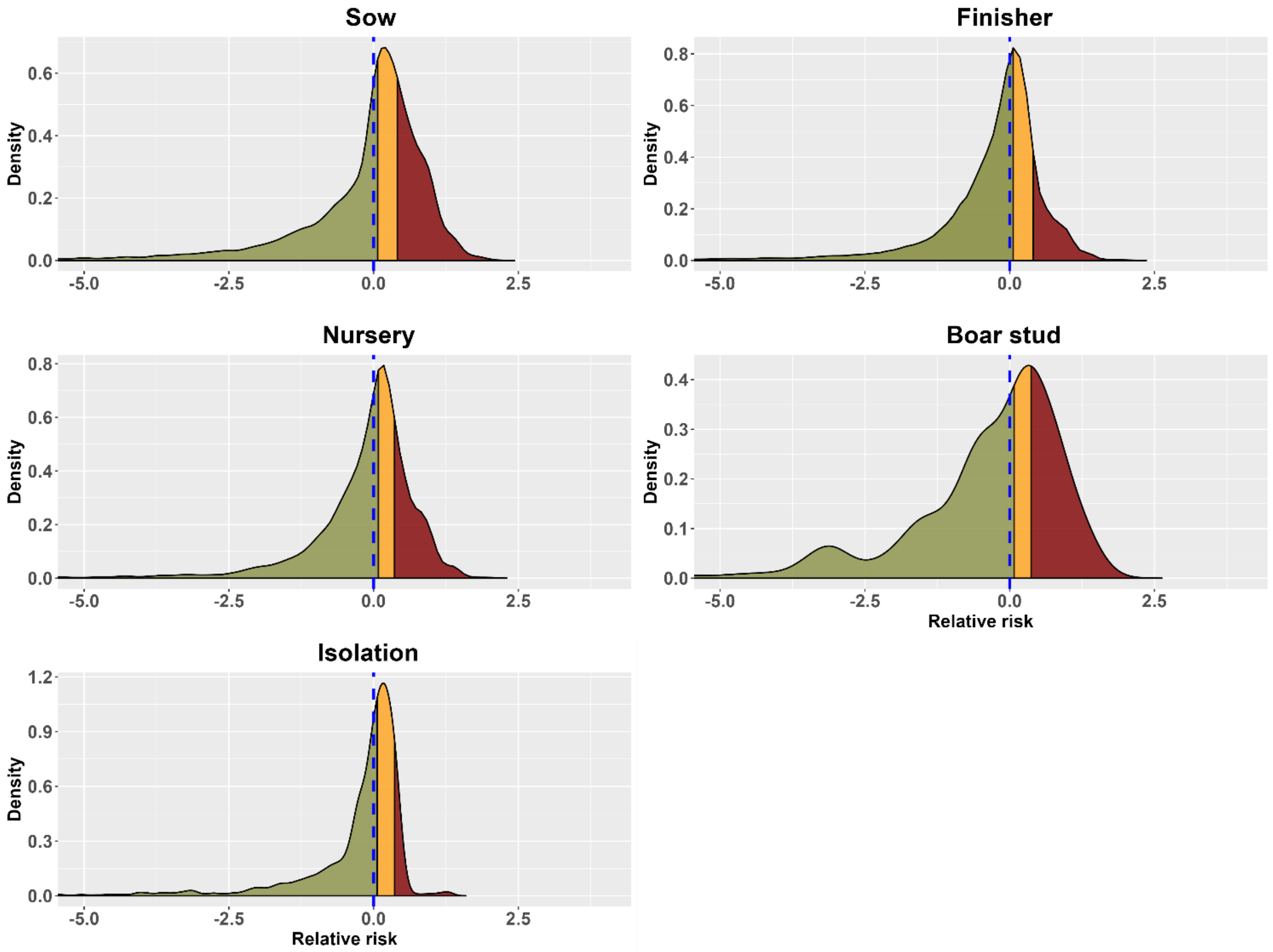

Supplementary Material Figure S9. Density plot of the spatiotemporal distribution of relative risk values for the year 2020.

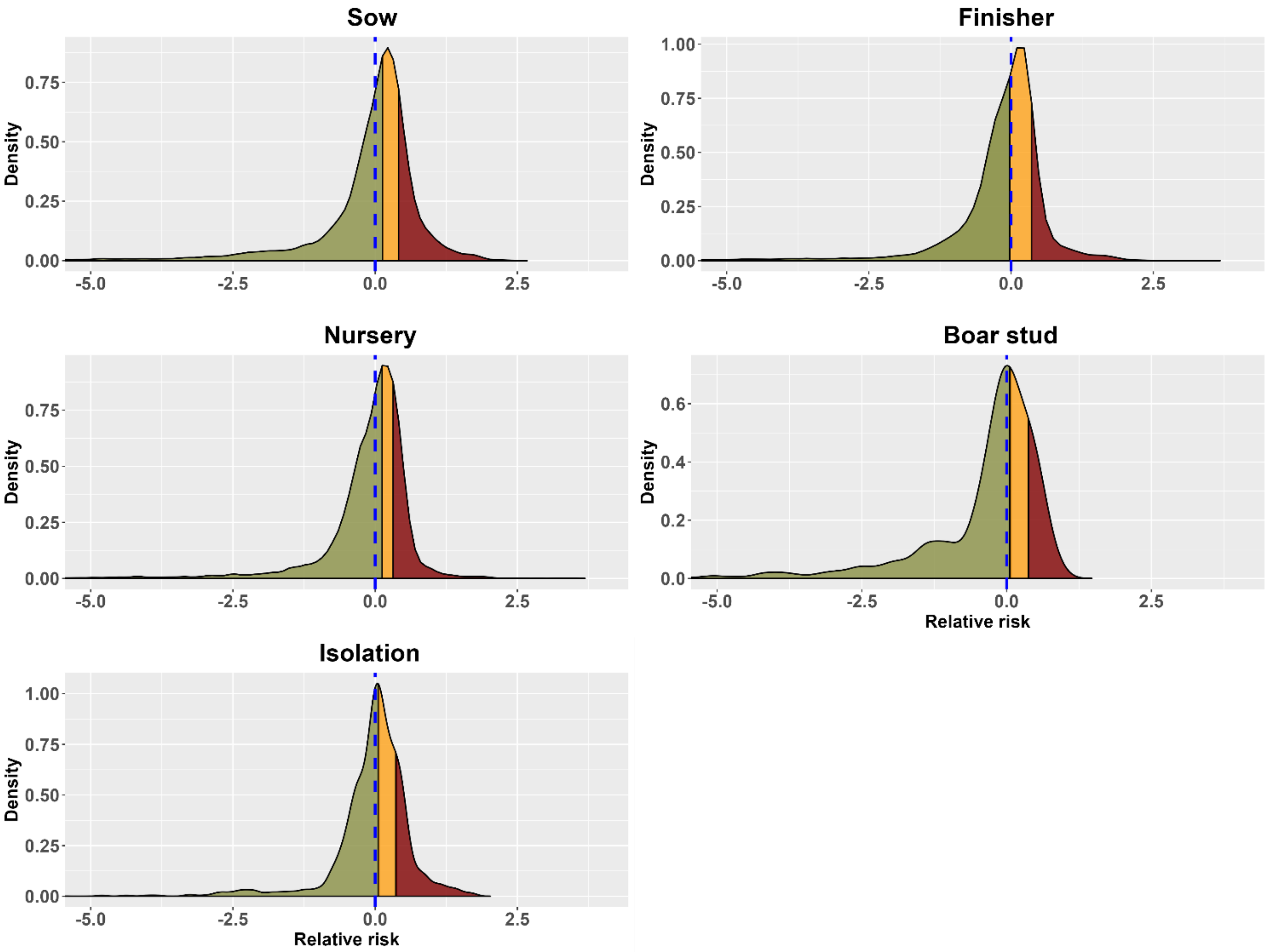

Supplementary Material Figure S10. Density plot of the spatiotemporal distribution of relative risk values for the 2017 - 2018 PRRSV season.

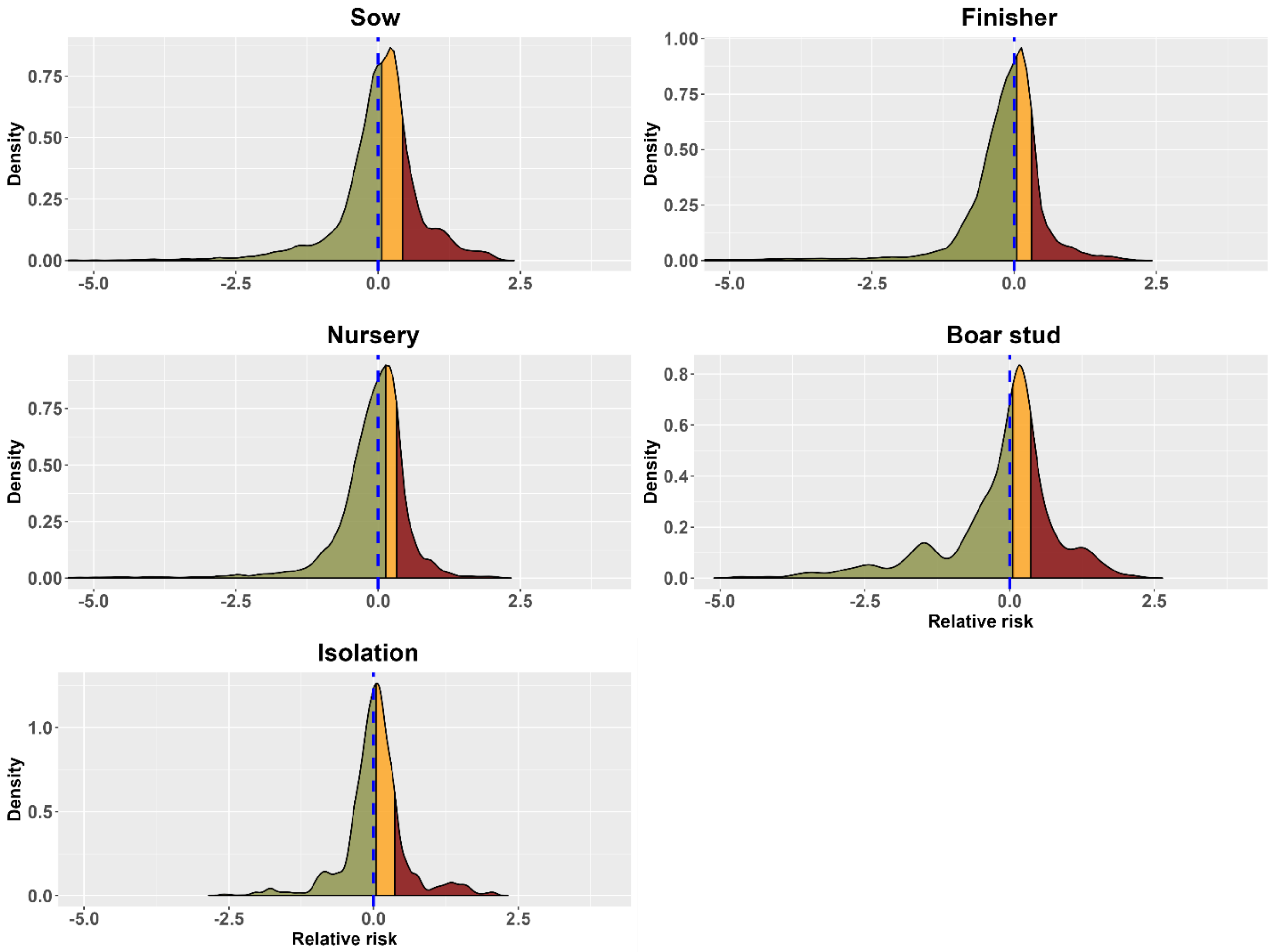

Supplementary Material Figure S11. Density plot of the spatiotemporal distribution of relative risk values for the 2018 - 2019 PRRSV season.

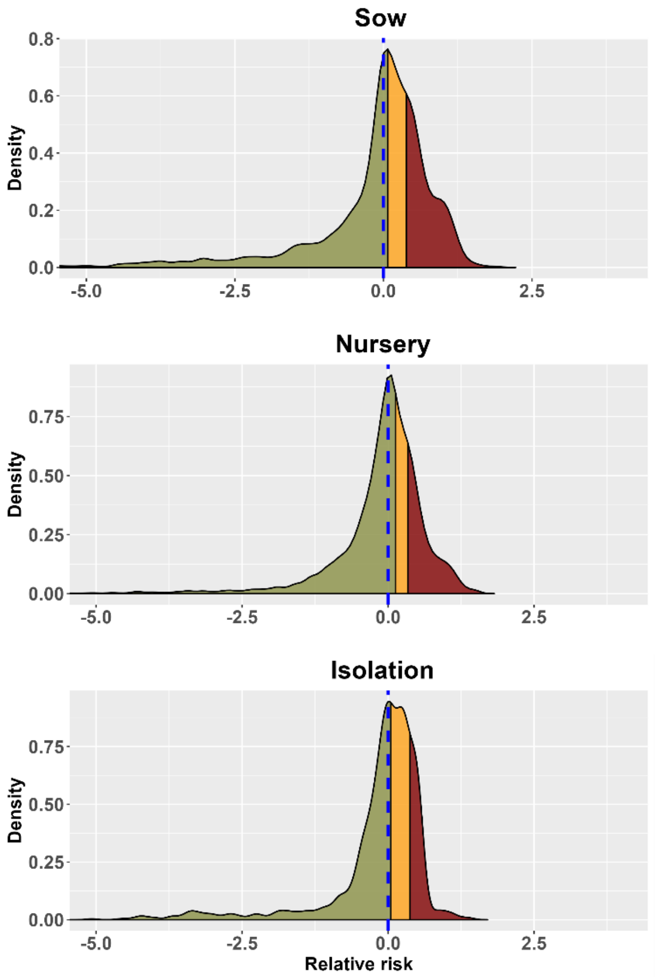

Supplementary Material Figure S12. Density plot of the spatiotemporal distribution of relative risk values for the 2019 - 2020 PRRSV season.

Supplementary Material Table S1. Description of variables considered in the Bayesian spatiotemporal hierarchical model.

Parameter	Definition	Summary Statistics (Min, Max, Median, IQR)	Reference
**Enhanced vegetation index (EVI)**	EVI data was downloaded at a 16-day temporal resolution and was used to calculate weekly averages by using one observation every 15 days. EVI was chosen over the Normalized Difference Vegetation Index since it corrects for some atmospheric conditions, canopy background	0.109, 0.781<br>0.397, 0.323 - 0.485	National Aeronautics and Space Administration (NASA), MODIS Land Products (Huete et al., 2002)

	noise, and is more sensitive in areas with dense vegetation. EVI values range from 0 to 1.		
**Aboveground biomass density**	Global Ecosystem Dynamics Investigation (GEDI) Level 4 B products offer estimates of aboveground biomass density in megagrams per hectare (Mg/ha) at a 1km x 1km resolution.	0.887, 64.675<br>3.032, 2.305 - 3.991	Oak Ridge National Laboratory, Distributed Active Archive Center for Biogeochemical Dynamics website (ORNL DAAC, 2022)
**Canopy height**	Global Ecosystem Dynamics Investigation (GEDI) Level 3 data provided as an average (meters) of the received waveform signal that was first reflected off the canopy (canopy height) relative to the WGS84 reference ellipsoid.	2.560, 9.336<br>4.691, 4.332 - 5.095	Oak Ridge National Laboratory, Distributed Active Archive Center for Biogeochemical Dynamics website (ORNL DAAC, 2022)
**Land surface elevation**	Global Ecosystem Dynamics Investigation (GEDI) Level 3 data provided as an average (meters) of the lowest received waveform (land surface elevation) signal received relative to the WGS84 reference ellipsoid.	4.233, 840.782<br>179.121, 168.644 - 189. 956	Oak Ridge National Laboratory, Distributed Active Archive Center for Biogeochemical Dynamics website (ORNL DAAC, 2022)
**Number of days temperature was between 4°C and 10°C (T[4°C,10°C])**	Daily minimum and maximum land surface temperature was used to calculate the daily average land surface temperature following the methodology described in Spangler et al., 2019.	42, 183<br>55, 53 - 58	Daymet: Daily Surface Weather Data (Thornton et al., 2020)
**Number of days relative humidity was below %20 (RH < 20%)**	Daily water vapor pressure data were used to calculate daily average relative humidity following methodology described in Spangler et al., 2019.	61, 273<br>76 - 79	Daymet: Daily Surface Weather Data (Thornton et al., 2020)

**In-degree**	Number of contacts with direction to a specific farm.	0, 97<br>3, 2 - 4	(Wasserman and Faust, 1994)
**Out-degree**	Number of contacts originating from a specific farm.	0, 41<br>0, 0 - 7	(Wasserman and Faust, 1994)
**Closeness centrality**	Closeness centrality measures how many steps are required to access every other vertex from a given node; this measure can be directed at incoming steps and otherwise outgoing steps.	0, 0.3<br>0, 0.0007 - 0.0008	(Freeman, 1978)
**PageRank**	Google Page Rank is a link analysis algorithm that produces a ranking of importance of all the municipalities of a network with a range of values between zero and one.	0.0002, 0.02<br>0.0003, 0.0002 - 0.0004	(Brin and Page, 1998)
**Betweenness**	Describes if the nodes and betweenness of edges are (roughly) defined by the number of geodesics (shortest paths) going through a vertex or an edge.	0, 320<br>1, 0 - 163	(Freeman, 1978)
**Clustering coefficient**	Measures the degree to which nodes in a network tend to cluster together, with a range of values between zero and one.	0, 1<br>0, 0 - 0.010	(Watts and Strogatz, 1998)
**Line of separation access points (LOSAP)**	On-farm biosecurity features representing locations where people or animals cross to gain access to buildings housing animals, and all other areas where employees and equipment have been completely sanitized. Supplementary Material Figure S17.	1, 49<br>5, 3 - 9	Rapid Access Biosecurity app™ (Machado et al., 2023)

**Perimeter buffer area access points (PBAAP)**	On-farm biosecurity feature locations where people or animals can gain access to the perimeter buffer areas (PBA), which may encompass the entire site or buildings housing animals and may or may not include feed bins. Equipment and vehicles should be sanitized prior to entry. Supply drop off area and carcass disposal locations may also be located in the PBA. Supplementary Material Figure S17.	1, 46<br>5, 4 - 7	Rapid Access Biosecurity app™ (Machado et al., 2023)
**Site entry (SE)**	Represents the location of entry into farm. Supplementary Material Figure S17.	1, 2<br>1, 1 - 1	Rapid Access Biosecurity app™ (Machado et al., 2023)
**Pig density**	Information about a farm's pig capacity was used as a surrogate for pig density.	160, 32,000<br>3,442, 2412 - 5,200	Morrison Swine Health Monitoring Project (MSHMP) (Perez et al., 2019)
**Farm density**	Variable created by applying a 17 km buffer around a farm's location and producing a count of farms within the buffer.	1, 305<br>116, 34 - 195	Extracted using farm location provided by the Morrison Swine Health Monitoring Project (MSHMP) (Perez et al., 2019)

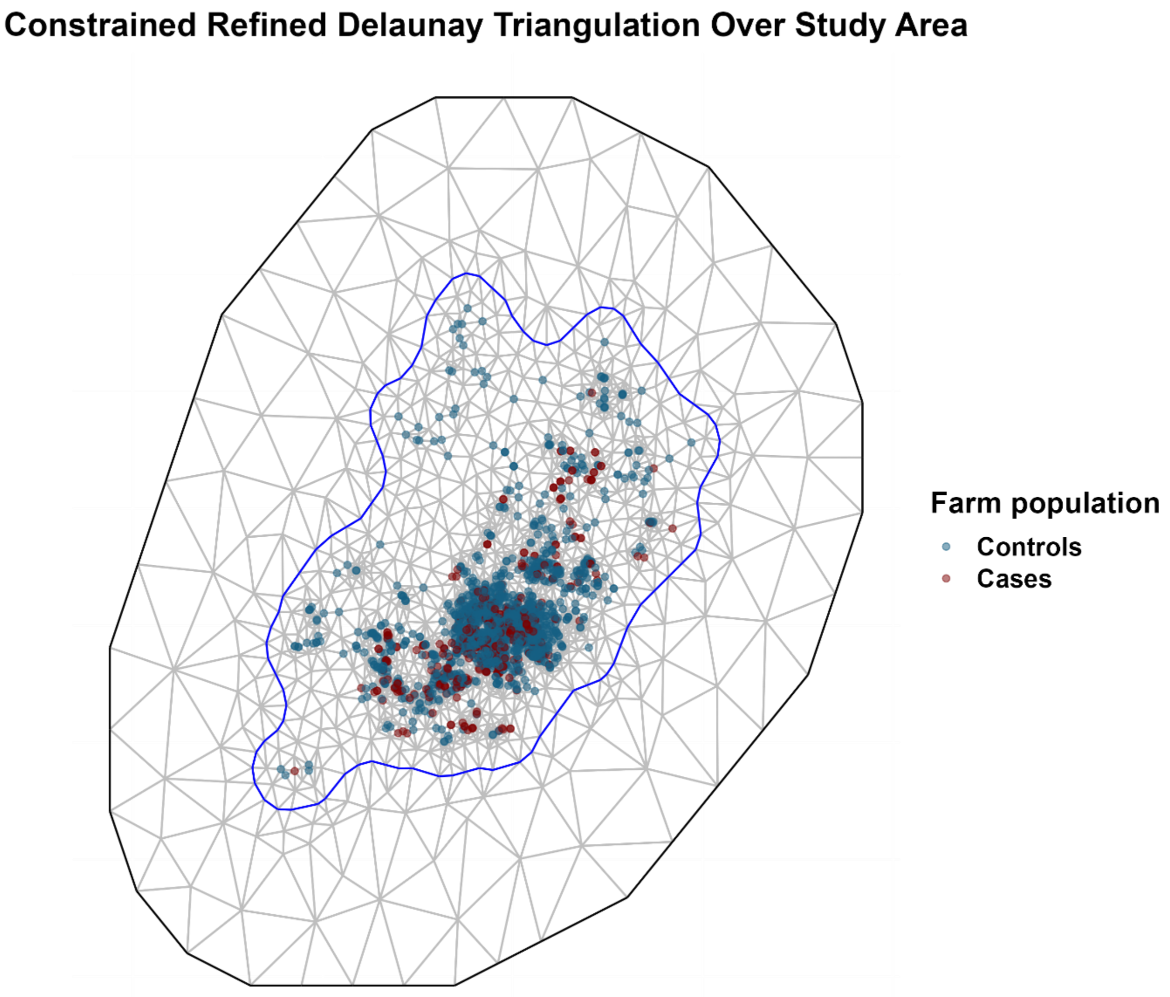

Supplementary Material Figure S13. Constrained refined Delaunay triangulation over the study area in 2020 with cases (red) and controls (blue) for the entire farm population (n = 1,948).

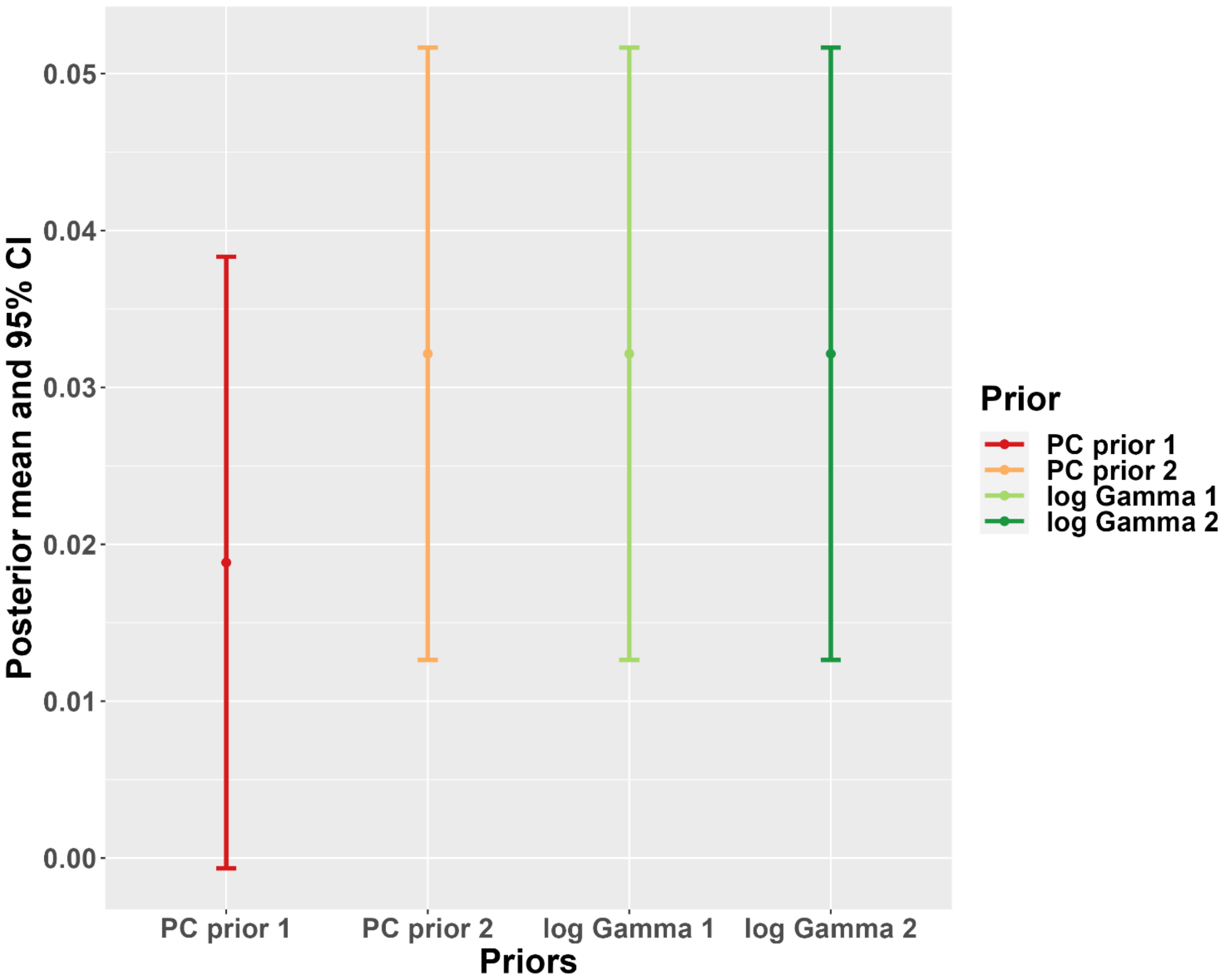

Supplementary Material Figure S14. Posterior mean and 95% confidence intervals (CI) for the four priors tested in the sensitivity analysis. All the CI overlap; therefore, the priors do not have a significant impact on the model.

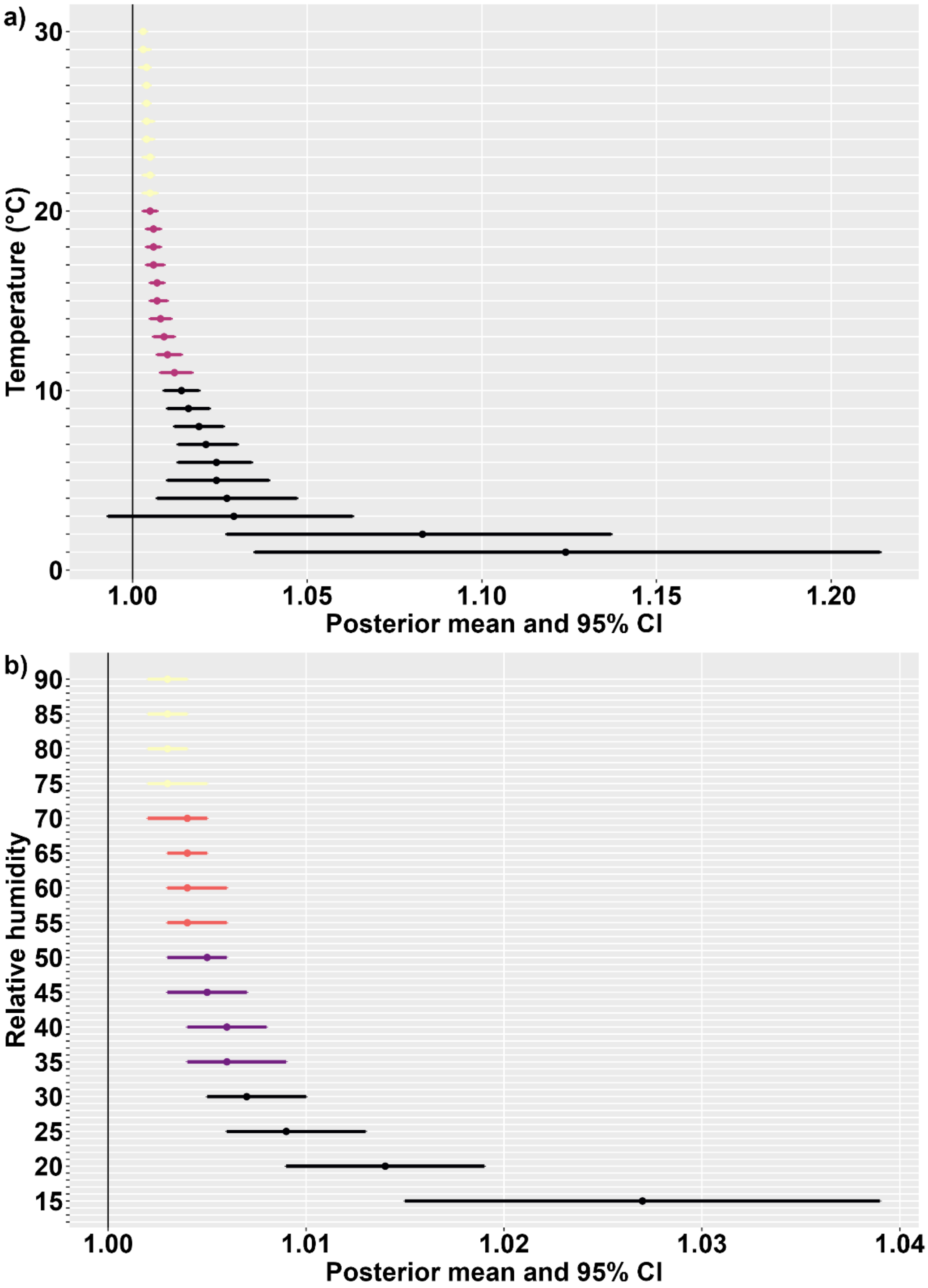

Supplementary Material Figure S15. Posterior mean and 95% credible intervals of temperatures (**a**) from 0° C to 30° C and relative humidity (**b**) from 0 to 90%. The Bayesian spatiotemporal hierarchical model was run sequentially with individual temperatures (0° C to 30° C) and individual relative humidities (0 to 90%). Lower temperatures (< 10° C) and relative humidity values (< 20%) represent values with stronger associations with PRRSV outbreaks as opposed to higher temperatures and relative humidity.

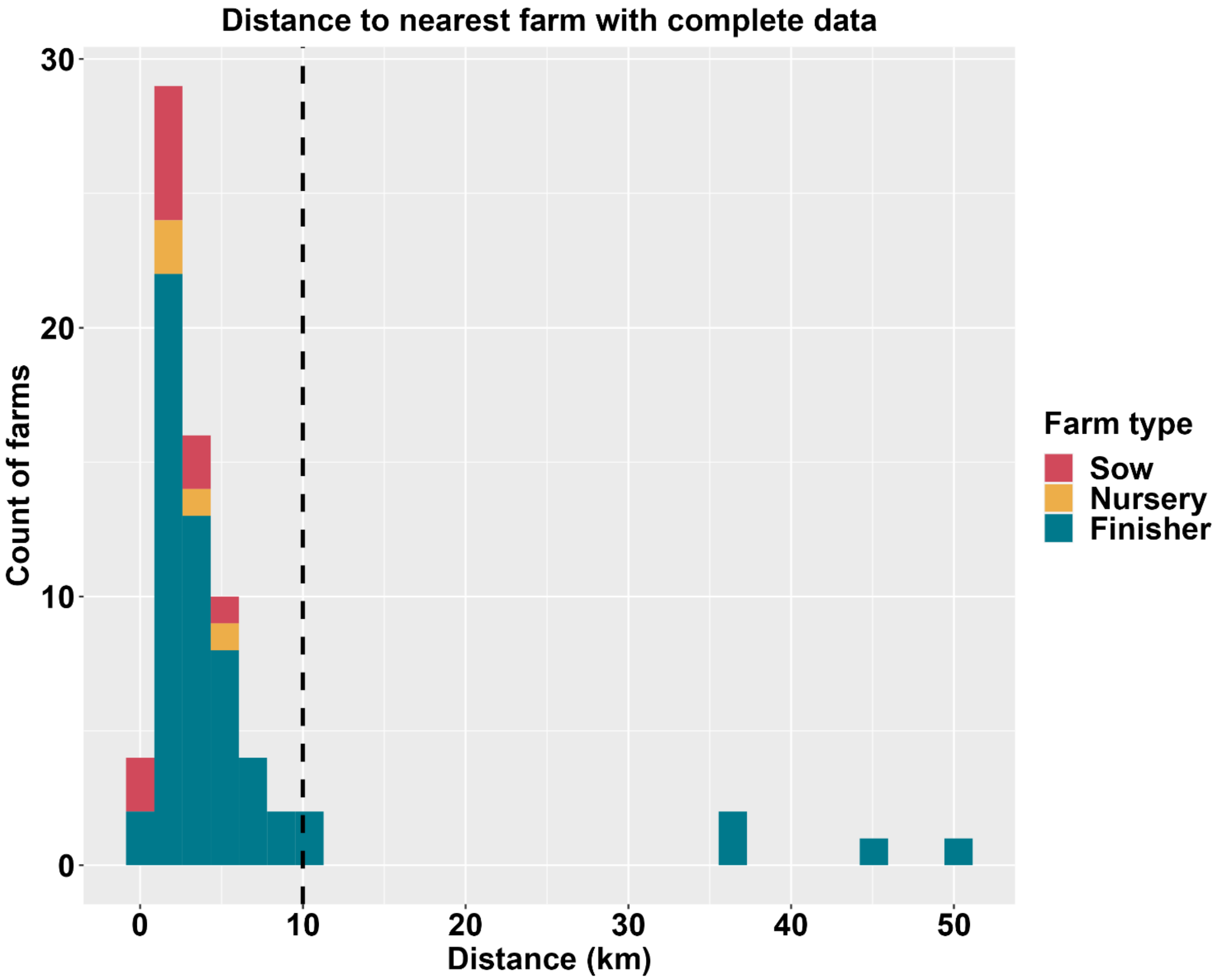

Supplementary Material Figure S16. Average distance (km) of the nearest farm with complete data used to fill in missing above-ground biomass density, canopy height, and elevation of 67 out of 71 farms missing data. Four farms exceeded the 10 km cut-off distance. The dashed black line represents the cut-off of acceptable distance to use.

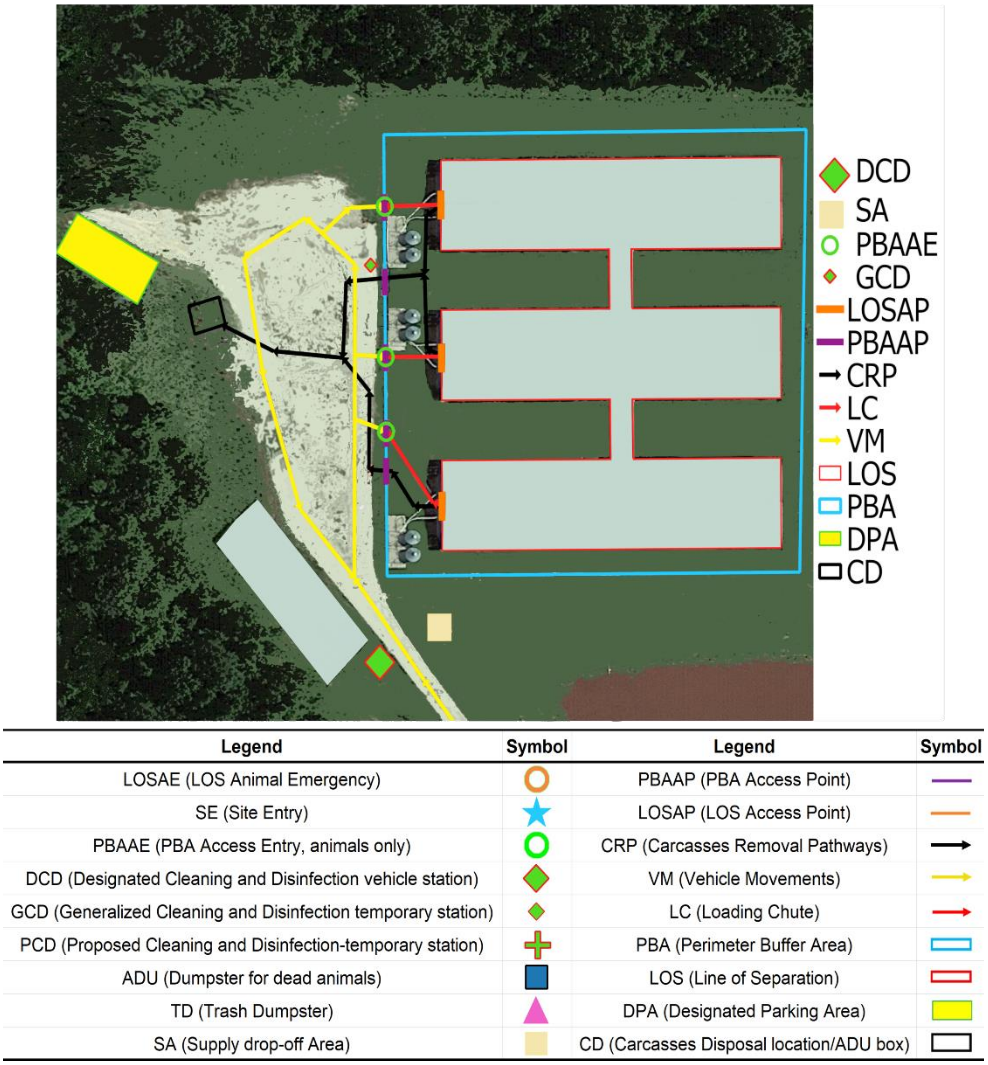

Supplementary Material Figure S17. Map of an example farm within RABapp™ (Machado et al., 2023) showing farm features including Site Entry (blue star), Line of Separation Access Point (LOSAP - orange line), and Perimeter Buffer Area Access Point (PBAAP - purple line).

Supplementary Material Table S2. PRRSV season count of cases and controls by farm type within significant ($p < 0.05$) high risk areas estimated using a spatial asymmetric adaptive smoothing approach for 2,293 farms (n = number of farms per farm type) in a dense pig production region of the U.S.

**PRRSV Season**	**Sow (n = 319)**		**Nursery (n = 468)**		**Finisher (n = 1458)**		**Isolation (n = 33)**		**Boar Stud (n = 15)**	
	Cases	Controls	Cases	Controls	Cases	Controls	Cases	Controls	Cases	Controls

**PRRSV Season 2017 - 2018**	13	24	14	40	11	55	0	0	0	0
**PRRSV Season 2018 - 2019**	28	45	5	69	6	146	0	7	0	2
**PRRSV Season 2019 - 2020**	35	35	6	73	9	134	0	7	0	2

Supplementary Material Table S3. Yearly count of cases and controls by farm type within significant ($p < 0.05$) high risk areas estimated using a spatial symmetric adaptive smoothing approach for 2,293 farms (n = number of farms per farm type) in a dense pig production region of the U.S.

**Year**	**Sow (n = 319)**		**Nursery (n = 468)**		**Finisher (n = 1458)**		**Isolation (n = 33)**		**Boar Stud (n = 15)**	
	Cases	Controls	Cases	Controls	Cases	Controls	Cases	Controls	Cases	Controls
**2018**	45	56	14	86	26	146	0	1	0	3
**2019**	17	15	2	30	2	25	0	0	0	0
**2020**	45	45	7	85	5	127	0	4	0	3

Supplementary Material Table S4. PRRSV seasons count of cases and controls by farm type within significant ($p < 0.05$) high risk areas estimated using a spatial symmetric adaptive smoothing approach for 2,293 farms (n = number of farms per farm type) in a dense pig production region of the U.S.

**PRRSV Season**	**Sow (n = 319)**		**Nursery (n = 468)**		**Finisher (n = 1458)**		**Isolation (n = 33)**		**Boar Stud (n = 15)**	
	Cases	Controls	Cases	Controls	Cases	Controls	Cases	Controls	Cases	Controls
**PRRSV Season 2017 - 2018**	18	29	15	48	10	73	0	1	0	0

**PRRSV Season 2018 - 2019**	7	13	1	27	2	27	0	0	0	0
**PRRSV Season 2019 - 2020**	41	42	10	89	10	168	0	10	0	2

Supplementary Material Table S5. Percent of high, medium, and low PRRSV risk levels (median and interquartile range (IQR)) based on weekly risk estimates obtained from the spatiotemporal analysis by farm type for each PRRSV season.

**PRRSV Season**	**Sow**			**Nursery**			**Finisher**			**Isolation**			**Boar Stud**		
	High	Med.	Low	High	Med.	Low	High	Med.	Low	High	Med.	Low	High	Med.	Low
**PRRSV 2017 - 2018**	24 (23-35)	24 (19-31)	42 (41-51)	25 (21-27)	27 (21-31)	47 (45-54)	26 (23-29)	24 (20-28)	50 (50-52)	21 (13-23)	23 (18-39)	55 (34-67)	20 (13-20)	20 (13-33)	60 (53-73)
**PRRSV 2018 - 2019**	37 (32-39)	15 (12-26)	45 (39-50)	19 (18-21)	15 (11-33)	65 (45-71)	16 (15-17)	15 (8-34)	69 (49-76)	12 (12-17)	24 (9-58)	67 (12-85)	33 (27-40)	20 (13-27)	47 (40-53)
**PRRSV 2019 - 2020**	26 (25-27)	22 (18-25)	51 (48-55)	19 (16-21)	20 (17-24)	60 (56-64)	18 (15-22)	19 (16-22)	63 (56-67)	30 (24-42)	33 (25-39)	33 (27-36)	33 (27-33)	20 (13-20)	47 (47-53)
**PRRSV Seasons***	29 (24-36)	22 (15-26)	47 (41-53)	21 (18-24)	22 (15-30)	58 (47-64)	18 (16-25)	20 (14-27)	56 (50-68)	21 (12-30)	30 (17-39)	36 (27-67)	27 (20-33)	20 (13-27)	53 (47-60)

* Median and IQR for all PRRSV seasons combined

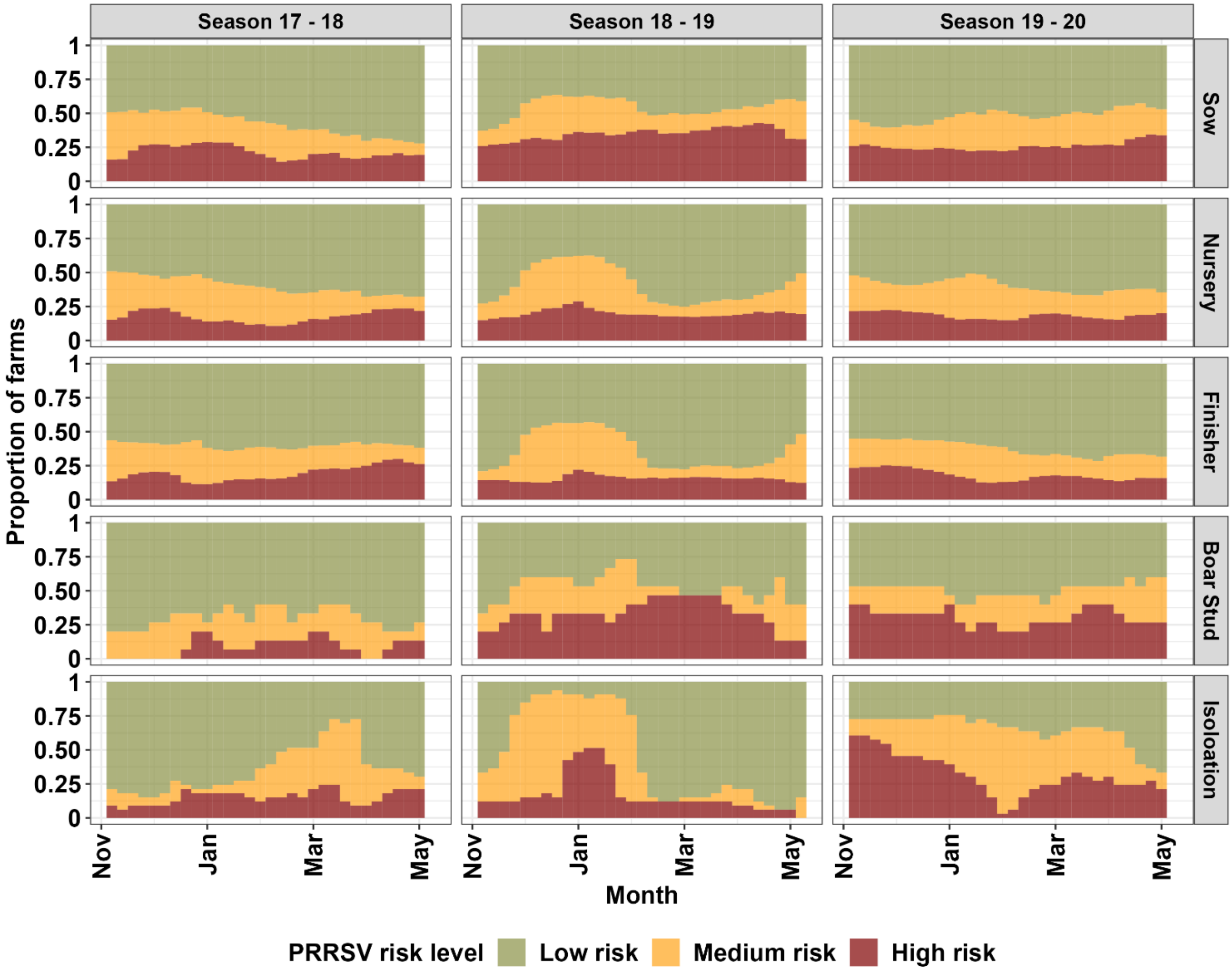

Supplementary Material Figure S18. Farm type breakdown of high, medium, and low PRRSV risk levels for the entire farm population (2,293) based on a 60% exceedance risk threshold for a) each week (1 - 30 weeks) in the PRRSV seasonal classifications.

Supplementary Material Table S6. Annual percentages of high, medium, and low priority indices (PI) by farm type. PI is calculated as an ordered percentage based on the relative risk value of a farm in reference to the maximum relative risk value of the entire farm population. High, medium, and low categories are then classified by quantile classifications.

Year	Sow			Nursery			Finisher			Isolation			Boar Stud		
	High	Med.	Low	High	Med.	Low	High	Med.	Low	High	Med.	Low	High	Med.	Low
**2018**	7	20	73	3	14	83	4	16	80	7	15	78	5	16	79
**2019**	14	32	54	5	28	67	7	26	66	8	27	65	25	37	39
**2020**	12	31	57	7	27	66	8	21	72	1	13	86	14	34	52

Supplementary Material Table S7. PRRSV season percentages of high, medium, and low priority indices (PI) by farm type. PI is calculated as an ordered percentage based on the relative risk value of a farm in reference to the maximum relative risk value of the entire farm population. High, medium, and low categories are then classified by quantile classifications.

PRRSV Season	Sow			Nursery			Finisher			Isolation			Boar stud		
	High	Med.	Low	High	Med.	Low	High	Med.	Low	High	Med.	Low	High	Med.	Low
**PRRSV 2017 - 2018**	1	5	94	0.2	4.8	95	2	7	91	0.6	4.4	95	0	7	93
**PRRSV 2018 - 2019**	9	29	62	6	31	63	8	34	58	13	36	51	17	31	52
**PRRSV 2019 - 2020**	3	13	84	1	8	91	2	10	88	1	8	91	1 `	16	83

S2. Network analysis

A total of 55,623 pig movements were used to construct a directed static network of pig movements for the year 2020 and was comprised of 2,286 vertices and 9,111 edges for a total of 104,624,297 pigs moved. An average of 1,881 pigs were moved per shipment.

Supplementary Material Table S8. Median and interquartile range (IQR) of node-level metrics calculated for the static pig network by farm type and cases and controls.

	**Sow**		**Finisher**		**Nursery**		**Isolation**		**Boar Stud**	
	Cases	Controls	Cases	Controls	Cases	Controls	Cases	Controls	Cases	Controls
**Degree**	8.5 (5 - 13)	9.5 (6 - 16)	6 (3 - 12)	3 (2 - 5)	11 (8 - 14)	10 (7 - 14)	-	5 (4.2 - 6.8)	-	2 (2 - 3)
**In-degree**	3 (1 - 4)	2 (1 - 4)	4 (3 - 11)	3 (2 - 4)	2 (1 - 4)	2 (1 - 3)	-	5 (2 - 5)	-	2 (2 - 3)
**Out-degree**	6 (4 - 10)	6 (3 - 10)	0 (0 - 1)	0 (0 - 1)	9 (7 - 11)	8 (6 - 11)	-	1 (0 - 1)	-	0 (0)
**Closeness centrality**	0.0000847 (0.0000633 - 0.0000910)	0.0000856 (0.0000788 - 0.0000907)	0.0000692 (0.0000649 - 0.0000930)	0.0000727 (0.0000649 - 0.0000804)	0.0000774 (0.0000718 - 0.0000837)	0.0000776 (0.0000718 - 0.0000841)	-	0.0000575 (0.0000574 - 0.0000575)	-	0.0000678 (0.0000678 - 0.0000678)
**Betweenness**	361 (118 - 731)	318 (107 - 804)	0 (0 - 2)	0 (0)	186 (120 - 340)	128 (53 - 241)	-	0 (0 - 126)	-	0 (0)
**Clustering Coefficient**	0 (0 - 0.018)	0 (0 - 0.04)	0 (0 - 0.01)	0 (0)	0 (0 - 0.018)	0 (0 - 0.015)	-	0 (0 - 0.003)	-	0 (0 - 0.17
**Page Rank**	0.000309 (0.000272 - 0.000403)	0.000304 (0.000268 - 0.000359)	0.000365 (0.000321 - 0.000517)	0.000329 (0.000292 - 0.000378)	0.000333 (0.000310 - 0.000420)	0.000319 (0.000281 - 0.000377)		0.000292 (0.000261 - 0.000298)	-	0.000265 (0.000258 - 0.000305)

References

Brin, S., and L. Page, 1998: The anatomy of a large-scale hypertextual Web search engine. *Comput. Netw. ISDN Syst.* **30**, 107–117, DOI: 10.1016/S0169-7552(98)00110-X.

Davies, T.M., and M.L. Hazelton, 2010: Adaptive kernel estimation of spatial relative risk. *Stat. Med.* **29**, 2423–2437, DOI: 10.1002/sim.3995.

Davies, T.M., K. Jones, and M.L. Hazelton, 2016: Symmetric adaptive smoothing regimens for estimation of the spatial relative risk function. *Comput. Stat. Data Anal.* **101**, 12–28, DOI: 10.1016/j.csda.2016.02.008.

Davies, T.M., and A.B. Lawson, 2019: An evaluation of likelihood-based bandwidth selectors for spatial and spatiotemporal kernel estimates. *J. Stat. Comput. Simul.* **89**, 1131–1152, DOI: 10.1080/00949655.2019.1575066.

Davies, T.M., J.C. Marshall, and M.L. Hazelton, 2018: Tutorial on kernel estimation of continuous spatial and spatiotemporal relative risk: Spatial and spatiotemporal relative risk. *Stat. Med.* **37**, 1191–1221, DOI: 10.1002/sim.7577.

Freeman, L.C., 1978: Centrality in social networks conceptual clarification. *Soc. Netw.* **1**, 215–239, DOI: 10.1016/0378-8733(78)90021-7.

Huete, A., K. Didan, T. Miura, E.P. Rodriguez, X. Gao, and L.G. Ferreira, 2002: Overview of the radiometric and biophysical performance of the MODIS vegetation indices. *Remote Sens. Environ.* **83**, 195–213, DOI: 10.1016/S0034-4257(02)00096-2.

Keeling, M.J., M.E. Woolhouse, D.J. Shaw, L. Matthews, M. Chase-Topping, D.T. Haydon, S.J. Cornell, J. Kappey, J. Wilesmith, and B.T. Grenfell, 2001: Dynamics of the 2001 UK foot and mouth epidemic: stochastic dispersal in a heterogeneous landscape. *Science* **294**, 813–817.

Lawson, A.B., and H. Zhou, 2005: Spatial statistical modeling of disease outbreaks with particular reference to the UK foot and mouth disease (FMD) epidemic of 2001. *Prev. Vet. Med.* **71**, 141–156.

Machado, G., J. Galvis, A. Freeman, F. Sanchez, C. Fleming, X. Hong, K. Mills, A. Sykes, A. Valencio, and D. Ebling, 2023: The Rapid Access Biosecurity (RAB) app™ Handbook. DOI: 10.17605/OSF.IO/Z5WBJ.

ORNL DAAC, 2022: ORNL DAAC for Biogeochemical Dynamics [Online] Available at https://daac.ornl.gov/ (accessed June 11, 2022).

Perez, A.M., D.C.L. Linhares, A.G. Arruda, K. Van Der Waal, G. Machado, C. Vilalta, J.M. Sanhueza, J. Torrison, M. Torremorell, and C.A. Corzo, 2019: Individual or common good? Voluntary data sharing to inform disease surveillance systems in food animals. *Front. Vet. Sci.* **6**, DOI: 10.3389/fvets.2019.00194.

Prince, M.I., A. Chetwynd, P. Diggle, M. Jarner, J.V. Metcalf, and O.F. James, 2001: The geographical distribution of primary biliary cirrhosis in a well-defined cohort. *Hepatology* **34**, 1083–1088.

Spangler, K.R., K.R. Weinberger, and G.A. Wellenius, 2019: Suitability of gridded climate datasets for use in environmental epidemiology. *J. Expo. Sci. Environ. Epidemiol.* **29**, 777–789, DOI: 10.1038/s41370-018-0105-2.

Thornton, M.M., R. Shrestha, Y. Wei, P.E. Thornton, S. Kao, and B.E. Wilson, 2020: Daymet: Daily Surface Weather Data on a 1-km Grid for North America, Version 4. DOI: 10.3334/ORNLDAAC/1840.

Wasserman, S., and K. Faust, 1994: Social Network Analysis: Methods and Applications, 1st edn. Cambridge University Press.

Watts, D.J., and S.H. Strogatz, 1998: Collective dynamics of ‘small-world’ networks. *Nature* **393**, 440–442, DOI: 10.1038/30918.
